# Blackbox error management: how do practices deal with critical incidents in everyday practice? A qualitative interview study

**DOI:** 10.1186/s12875-023-02206-2

**Published:** 2023-11-29

**Authors:** Aljoscha Bodek, Marina Pommée, Alexandra Berger, Maria Giraki, Beate Sigrid Müller, Dania Schütze

**Affiliations:** 1 Institute of General Practice, Goethe University Frankfurt, Theodor-Stern-Kai 7, 60590 Frankfurt Am Main, Germany; 2 Association of Statutory Health Insurance Physicians Westphalia-Lippe, Robert-Schimrigk-Str. 4-6, 44141 Dortmund, Germany; 3 Frankfurt Reference Centre for Rare Diseases, University Hospital of the Goethe University Frankfurt, Theodor Stern-Kai 7, 60590 Frankfurt, Germany; 4 Department of Operative Dentistry, Center for Dentistry and Oral Medicine (Carolinum), Goethe University Frankfurt, Theodor-Stern-Kai 7, 60596 Frankfurt Am Main, Germany; 5 Faculty of Medicine and University Hospital Cologne, Institute of General Practice, University of Cologne, Kerpener Str. 62, 50937 Cologne, Germany

**Keywords:** Error management, Patient safety, Ambulatory care, Incident, Reporting, Qualitative research

## Abstract

**Background:**

Error management plays a key role in patient safety. It is a systematic approach aimed at identifying and learning from critical incidents by reporting, documenting and analyzing them. Almost nothing is known about the incidents physicians in outpatient care consider to be critical and how they deal with them. We carried out an interview study to explore outpatient physicians’ views on error management, discover what they regard as critical incidents, and find out how error management is put into practice in ambulatory care.

**Methods:**

We conducted 72 semi-structured interviews with physicians from ambulatory practices. We asked participants what they considered to be a critical incident, how they reacted following an incident, how they discussed incidents with their coworkers, and whether they used critical incident reporting systems. The interviews were transcribed verbatim and analyzed using qualitative content analysis.

**Results:**

Interviewed physicians defined the term “critical incident” differently. Most participants reported that they recorded information on incidents and discussed them in their teams. Several physicians reported taking a ‘pay better attention next time-approach’ to the analysis of incidents. Systematic error management involving incident documentation, analysis, preventive measure development, and follow-up, was the exception.

**Conclusions:**

To promote error management, medical training should include teaching on the topic, so that medical professionals can learn about critical incidents and how to deal with them in an open and structured manner. This would help establish the culture of safety that has long been called for internationally.

**Supplementary Information:**

The online version contains supplementary material available at 10.1186/s12875-023-02206-2.

## Background

Patient safety discussions focus increasingly on outpatient care [1–3]. In Germany, most physician-to-patient contacts occur in the outpatient sector [4]. The sector consists of more than 68,000, mostly small practices, with an overall average of 2.0 physicians and 5.2 health care assistants per practice [5]. Consultations in German primary care last an average of 8 min [6]. The majority of practices focus on family medicine, but other highly specialized practices such as outpatient cardiology centers, and practices specializing in surgery, orthopedic medicine, dermatology and psychiatry are also common. Dental healthcare providers are also almost solely to be found in the outpatient sector. Error management plays a key role in patient safety, as errors occur in around two of 1.000 consultations [7–9]. In Germany, error management is recommended in both the outpatient and inpatient sectors in a quality management guideline (QM-RL) and is required by law [1]. Accordingly, practices must maintain "error management and error reporting systems", more details are not specified. Error management is a systematic approach aimed at identifying and learning from critical incidents, errors and near misses, in order to prevent them from happening again [1]. Such incidents can be reported and documented voluntarily and anonymously, and should not involve any threat of sanctions for the reporting person. Incidents should be analyzed and discussed, and preventive measures should be developed and followed up upon. A critical incident can be defined as "an incident that increases the risk of a serious adverse event or that actually results in a serious adverse event [10]. An adverse event, in turn, is "an unintended negative outcome that results from the treatment rather than the disease" [10]. Some countries like Denmark and UK have mandatory error reporting systems also in primary care in place, but international studies on how practices really deal with error management are scarce [11, 12]. Especially for German outpatient care, almost nothing is known about how doctors define critical incidents or adverse events and which incidents they consider relevant, and how they deal with them. It is still largely unclear what internal structures are in place in practices, and how practice teams actually deal with events that (may) present a risk to patient safety.

We carried out this interview study with the aim of exploring outpatient physicians’ views on error management in medical practices, finding out how they define critical incidents, and how they conduct error management in their ambulatory practices.

## Methods

### Design

We conducted semi-structured interviews with specialists in one of five disciplines that worked in ambulatory practices. The ethics committee of the Goethe University Frankfurt approved the study on October 24, 2019 (ID: 19–413). The following presentation of methods and results follows the consolidated criteria for reporting qualitative research (COREQ) [13].

### Recruitment

Recruitment took place between March and September 2020 [14]. We contacted 1255 physicians that worked in one of five predefined medical specialties (general practice, dermatology, orthopedic surgery, psychiatry/psychotherapy, dental care). We used the publicly accessible physician registers of the Associations of Statutory Health Insurance Physicians to obtain physicians’ addresses. We also contacted relevant professional associations, which called on physicians to participate. Physicians were informed about the study by post or e-mail and invited to an interview. Interested physicians filled out a contact form and were contacted by telephone or e-mail to arrange an appointment.

We received 92 responses. It was not possible to arrange an interview appointment with nine of the 92 interested persons, and a further eleven persons did not fulfill the inclusion criteria for study participation because they were also affiliated with a hospital. As we were interested in the outpatient perspective on error management, we considered work in a hospital to be an exclusion criterion. Study participants received 50€ for taking part in an interview. All participants provided written informed consent.

### Data collection and interview guide

We conducted semi-structured interviews with 72 physicians between March and October 2020. The interviews were carried out by telephone, recorded, and transcribed verbatim. Interviewers also made notes on the atmosphere in which the conversations took place, their overall impressions, and on new topics that were raised. Participants were randomly assigned to one of the three interviewers: DS (female sociologist), MP (female master of public health) and ABo (male sociologist and medical student).

The interview guide was developed according to the national quality management guideline for practices and own previous work [1, 15–17]. It was presented to an internal interdisciplinary group of qualitative researchers, who discussed and revised it as necessary. It was also piloted in seven interviews with physicians from the target disciplines, after which further adjustments were made. The pilot interviews were not transcribed and analyzed. The final interview guide (see Additional file 1) contained questions on the following topics: Critical incidents in practices, responses to an incident, communication within the team, use of critical incident reporting systems (CIRS), recommendations for error management.

### Data analysis

Data analysis was carried out by DS, MP and ABo using qualitative content analysis [18], a procedure in which text passages are coded or indexed. The analysis of the data is done by a synoptic interpretative analysis of the passages that have certain categories and characteristics in common. The aim of this procedure is to form empirically meaningful categories and, on the basis of these, to be able to formulate structured statements about the phenomenon studied [14]. All transcripts were coded with the support of MAXQDA software. First, a code tree was created on the basis of an iterative process in which all three researchers coded the same interviews. Due to the wide range of views expressed in the interviews, researchers first re-sorted and classified the interview material by re-sorting it in rough terms and assigning text passages to the categories "Definition of critical incidents", "Realization of error management" and "Attitudes towards, and experiences with error management". Based on this initial rough data re-sorting, each interview was then summarized, and categories and sub-categories were developed inductively by the three researchers through discussion among each other. The categorization was then applied to all interviews. In an iterative process, the distinguishing sub-categories were revised and supplemented until they adequately represented the material [14]. Interim results and possible further steps were continuously discussed by the researchers and the project supervisor BSM. The approach to data analysis was also discussed in an interdisciplinary research group.

## Results

We interviewed 72 physicians, each of whom was a specialist in one of five fields (general practitioner, dermatologist, orthopedic surgeon, psychiatrist/psychotherapist, dentist). The interviews lasted from 8 to 55 min, and had an average duration of 25 min. Sample characteristics are described in Table 1. In the following, we present the main categories of our analysis and their different sub-categories, and describe how error management was realized in the participating medical practices. Since no distinct differences between the specialist groups emerged in the analysis, we present the totality of the interviewees in the results. Where differences between the groups were found, this is explicitly mentioned in the text. Each quotation is assigned a code: A = general practitioner, C = surgeon, orthopedic surgeon D = dermatologist, P = psychiatrist, Z = dentist and an individual number.

**Table 1:** Study participants

	***N* = 72**
**Sex**	
Female	35 (48.6%)
Male	37 (51.4%)
**Type of practice**	
Solo practice	33 (45.8%)
Joint practice	28 (38.9%)
Medical service center	6 (8.3%)
Group practice	5 (6.9%)
**Specialty**	
General practice	15 (20.8%)
Dermatology	15 (20.8%)
Orthopedic surgery	15 (20.8%)
Psychiatry/Psychotherapy	10 (13.9%)
Dentistry	17 (23.6%)
**Number of non-physician employees in practice (*n* = 70)**	
Mean (SD)	6.2 (4.5) (Min. 0; Max. 29)
**Number of physicians in practice**	
Mean (SD)	2.3 (1.6) (Min. 1; Max. 8)
**Professional experience as physician (in years) (*n* = 71)**	
Mean (SD)	24.9 (10.3) (Min. 3; Max. 44)
**Years working in current practice**	
Mean (SD)	12.9 (9.2) (Min. 1; Max. 32)
**Function in practice**	
Practice owner	58 (80.6%)
Physician employee	14 (19.4%)

### Understanding of the term “critical incident”

**Fig. 1:**
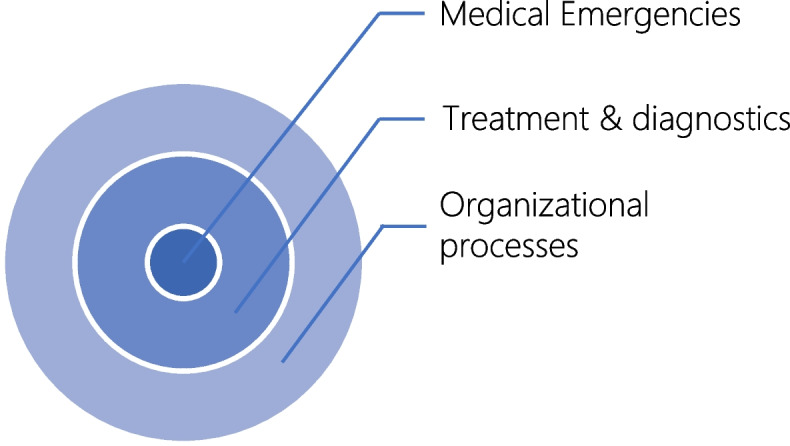
Layers of the definition of “critical incidents”

Participants interpreted the term “critical incident” in different ways. Most physicians included incidents that took place as part of an organizational process, and did not confine their understanding of a critical incident to events that occurred during treatment and diagnostic procedures, and/or that led to an emergency. With regard to organizational processes, physicians frequently mentioned errors in patient identification or in making appointments. Other respondents considered critical incidents to relate solely to treatment, diagnostic procedures and emergencies. Surgeons often mentioned operations in this regard, while psychiatrists referred to errors made during psychotherapeutic consultations. Some of the participating dentists considered critical incidents to be primarily medical emergencies, such as life-threatening situations for patients that require immediate action (e.g. cardiac arrest in response to a local anesthetic, or sudden heavy bleeding). Figure 1 illustrates the spectrum of the physicians’ understanding by different layers.

### Discussion of events

Participating physicians differed in the way they dealt with critical incidents in their practices and within their teams. In most practices, incidents were discussed at regular team meetings, which generally took place with a time lag to the incident. The intervals at which meetings were held differed from practice to practice. While in some cases they were held weekly, others held their team meetings monthly or quarterly. As error management was viewed as something that affected everyone in the practice, the meetings generally involved all employees (physicians and health care assistants). During the meetings, the whole team discussed the adverse events that had occurred, as well as possible reasons and preventive measures:

»And that’s where such things – problems and mistakes - are discussed and that’s where we, as a team, try to find solutions and ways of preventing them« (A11)

Some of them evaluated whether their measures actually worked in practice at a later meeting:

»And the following Wednesday, we discuss the topic again, and we ask the whole group: “Did things work out ok?” “What do the others think?” “Who noticed anything different?” “And where did things not go smoothly?”.« (D82)

In other practices, participants said spontaneous meetings generally took place shortly after an event. Everyone in the practice was told what had just happened or had almost happened, and if necessary, the practice owner gave instructions on how to proceed in the current situation. Events were addressed, rather than discussed and analyzed, with the main aim being to react quickly and find a (supposedly) quick solution.

»And then we all meet up in the kitchen […] that’s how we do it, we get together more or less straightaway, and then discuss it. We communicate quickly, basically immediately.« (A47)

»Then we discuss it afterwards. […] Ours is just a small practice. One or two health care assistants are there at most. So you generally find out about everything directly and can say, “no, please do it like this”, or “correct it by doing that”, or “call up again and rectify the situation”.« (A02)

Some interviewed doctors regarded error management solely as a means of responding to acute problems and medical emergencies, with the aim of reducing patient harm in the context of a specific event.

»In the case of one patient, who was taking hydromorphone, the wrong dose was written down. So she of course received twice the dose she should have. But that wasn’t noticed until she came back wanting a follow-up prescription, after having been to hospital in the meantime. Whether that had anything to do with the medication was difficult to ascertain. But of course we changed the follow-up prescription.« (O42)

»Yes, in our case, if we pull out the wrong tooth, for example, then it’s no problem, we just provide a dental implant free of charge. When we see it’s our mistake, then we try to correct it and to be fair about it.« (Z21)

These participants considered an incident to have been brought to an end when “the best possible result has been achieved”. (D20)

»Sometimes we can correct things that have gone wrong immediately. Then it’s all over, after the correction has been made.« (A02)

### Documentation of events

While some physicians said they did not document events in their practice at all, others had established a special procedure by which to do so. Terms such as a reporting system or CIRS (critical incident reporting system) were not used by the respondents. They also did not distinguish whether critical incidents with or only those without harm should be documented. The question was rather how events, errors or “anything else that came to mind” (D43) should be brought up and dealt with at a later meeting. The practices used three different methods of recording any issues they had had:

1. Incidents are documented at a central location to which every staff member has access, e.g. an electronic patient file dedicated to incidents (“Mr. Error”) or a blackboard in the common room.
2. Incidents are documented by staff members individually, e.g. by writing them down on paper at their desk.
3. One person is responsible for documenting incidents and everyone reports relevant events to that person.

### Types of preventive measures

Many participating physicians said that practice teams not only adopted immediate measures to deal with an acute event, but also introduced safety measures to prevent such an event from happening again. These preventive measures were sometimes system-oriented and sometimes person-oriented.

#### System-oriented measures

Some physicians reported that events were discussed in their practices, and that they focused on what could be “changed, adapted, improved” (A057). In these practices, causes and preventive measures were not usually sought in the actions of individuals, but in the design of processes and the system as a whole.

»Well, let’s say an erroneous prescription is issued – how can that be? Where in the process can there be a problem that makes that kind of mistake crop up? So we discuss the processes, and when you notice – Aha! It’s because of a certain click on the PC […] then that particular thing will be specified in the workflow accordingly.« (C70)

The endeavor to analyze events independently of individuals was emphasized by many participants.

»So when our Wednesday meeting begins with, perhaps, someone saying: "This and that didn’t go very well" And then someone else says: "Yes, who was it this time?" Then we immediately interrupt and say, "it makes no difference who it was. No one needs to know who it was. Let’s just ask ourselves why it happened and why we don’t want it to happen again."« (D82)

#### Person-oriented measures

Some participants indicated that they were looking for the person responsible for an incident. The staff member concerned, or the whole team, was then told to ‘pay more attention in the future’. The underlying cause of the problem or the contributing factors that led to it were not discussed. Responsibility was thus assigned to the individual.

»And if they do occur, then the person responsible for it, even if it’s me, is severely reprimanded. I would say: "Now listen, you know we can‘t let that happen." And then at our regular official meeting, it‘s simply mentioned again: "It’s ok, it happens, pay more attention next time".« (Z29)

Moreover, some participants said they dealt with adverse events on their own. Such persons primarily considered critical incidents to be either emergencies, or other adverse events that occurred when treating a patient, and tended to react to the incidents by seeking to improve their medical competence. They regarded their medical skills as the reason for and the solution to the incidents.

»When I notice that the quality of my work is declining, and I think it’s important that I am always my own harshest critic. That’s why I’m always taking part in further education and training programs. I learn the most from them and I accept new solutions and integrate them into my work.« (Z63)

### Participants’ safety culture

Most participants had an open attitude towards the topic of errors. They emphasized that everyone makes mistakes and that it is important not to conceal them. They stressed the importance of openness both towards patients and physician colleagues, as well towards other team members.

»Well everyone’s afraid of making mistakes, aren’t they? And you have to try to take that fear away from people, right? Because everyone makes mistakes, you can’t completely prevent them, and you have to learn to deal with them.« (C068)

»And being honest with the patients. That is the most difficult thing but also the most important thing. Most patients appreciate it.« (Z079)

At the same time, it became clear in the interviews that this openness does not automatically result in comprehensive error management.

»And no one discussed how it happened, and they just said, “no, that shouldn’t happen”. And they listened to what happened and had a positive attitude towards the whole thing, but in the implementation, there was always a reason why the implementation couldn’t be successful.« (A34)

Some physicians explicitly reported positive or negative experiences they had had in their professional careers that had shaped their attitude towards dealing with errors. While some participants were motivated by positive experiences, such as superiors dealing with errors in an open-minded manner without resorting to punitive sanctions, others reported negative examples that made them realize that they themselves would like to behave differently. They reported that blame is commonplace in clinical practice, and that the importance of error management and open communication was rarely taught to young physicians:

»And, well, I have worked in three practices and three, four or five clinics and, to be honest, I have rarely experienced anything else. That is to say in most clinics and practices, it was always the case that the first question was: “Who was it?”.« (D82)

»In medical training, the only thing you learn about is medicine, nothing else.« (D82)

## Discussion

This interview study was the first to provide an insight into error management in different kinds of German ambulatory practice. Most participating physicians were open to the idea of error management and reported that they discussed adverse events at regular team meetings. At the same time, a person-oriented ‘pay better attention next time-approach’ by which responsibility is shifted to the actions of the individual employee without looking for causes in the system and the underlying conditions, still seems to play a significant role. Overall, the interviews showed that systematic error management that included potential or actual critical incident documentation, analysis, the development of preventive actions, and follow-up measures, was rare.

Our study results show that the interviewed physicians differed in the way they defined the term “critical incident”. Establishing commonly shared definitions of key concepts is a precondition of comprehensive and systematic error management. As pointed out by other studies, the ability to identify an incident as critical is crucial if it is to be dealt with properly [19–23]. The use of heterogeneous terminology to describe the same incident is often the cause of missed opportunities to learn from and prevent such incidents in the future [24]. It is therefore important that medical professionals share an understanding of key concepts relating to error management if they are to prevent errors in the future. In this respect, it is important that guidelines like the German quality management guideline provide comprehensive and consistent definitions and thus helps establish a common basis for error management. Furthermore, health care professionals should be introduced to these concepts early in their careers, as recommended by the WHO and already practiced in several countries [25, 26].

In Germany though, health care professionals do not receive formal training in safety culture and dealing with critical events on a regular basis [20]. This shortcoming was reflected in our findings, with participating physicians attributing errors to individuals. Our findings also suggested that participating physicians were influenced early on in their careers by the attitudes of their supervisors towards error management and safety culture, particularly if they perceived them as setting a positive example. This emphasizes the importance of raising the general awareness of a culture of safety as early as possible in medical education [27]. Educational concepts have already been tested and should urgently be integrated into medical curricula in order to establish the safety culture that has long been demanded internationally [7, 27]. As pointed out by Mitchell et al., it is very important to establish a culture of safety and to link it to clearly defined responsibilities in ambulatory practices [8]. Furthermore, recent work has demonstrated that hierarchical structures are major obstacles to the implementation of structured error management in ambulatory practices, particularly when physicians are skeptical about the benefits of error management [16].

Our study results show that some participants blamed adverse events upon themselves and sought to prevent them in the future by improving their skills as physicians. Results from studies on diagnostic errors indicate that regretting certain diagnostic decisions can be momentous for physicians making them second-degree victims [28–30]. It is necessary to have independent contacts to whom physicians can turn in case of such events. In this way, they can learn from diagnostic or treatment errors and avoid the risk of practicing defensive medicine in the future [28–30].

In our study sample, most ambulatory practices used some form of basic internal error management system. The strategy of gathering data on incidents and storing it at a central location in the practice was common. In most cases, employees could voluntarily report potential or actual critical incidents anonymously or confidentially and in a low-threshold manner. Collecting information in this manner is similar to formally establishing an internal error reporting system, although participants do not explicitly label it as such. This provides a basis upon which to promote error management. Overall, many incident reports are already available in ambulatory practices, but they are currently only stored locally. This treasure trove of reports and preventive measures should be recorded digitally and shared, so that practices do not have to develop their own solutions and can learn from each other. In the future, broad and productive error management will require such reports, common definitions and a higher awareness of the impact and causes of critical incidents through courses at an early stage of medical education [8].

### Strengths and limitations

When interpreting the results, it should be borne in mind that participating practices are likely to have a greater-than-average interest in the topic of error management. The interviews took part during the first months of the COVID-19 pandemic. This made it easier to recruit dentists, for example, as they saw fewer patients and had more time to participate in the study. On the other hand, GP practices in particular were significantly more busy than before, [31] which suggests an even higher awareness of the topic of error management among participating GPs. It is therefore all the more relevant to note that even in these committed ambulatory practices there is still room for improvement in terms of structured error management. In order to get a first and broad view of error management in the outpatient sector, we did not focus on one specialty. We believe the reported approaches and attitudes provide a comprehensive insight into structures for sustainable error management in a diverse sample in terms of gender, specialty, and practice type. Future studies should build on our findings, e.g., using a quantitative approach, to detect differences in error management between specialties.

## Conclusions

The study results provide an insight in the current state of structured error management, as well as attitudes and perspectives, and thus serve as an important foundation for the development of strategies to promote error management in ambulatory care. To establish a culture of safety and to provide physicians with greater knowledge on how to develop and track strong error prevention measures, it is important to learn how to deal with incidents in an open and structured manner. Courses teaching such skills should be an integral part of medical training.

### Supplementary Information

**Additional file 1.**

## Data Availability

The datasets generated and/or analysed during the current study are not publicly available due to the confidentiality promised to respondents at the time of consent but are available from the corresponding author on reasonable request.

## Abbreviation

CIRS: Critical incident reporting system
