# Supplementary material for: Blackbox error management: how do practices deal with critical incidents in everyday practice? A qualitative interview study
Source: BMC Prim Care. 2023 Nov 29;24:251. doi: 10.1186/s12875-023-02206-2 (PMC10685626; doi:10.1186/s12875-023-02206-2)
Supplement: Supplementary file 1 — Additional file 1. [file 12875_2023_2206_MOESM1_ESM.pdf]

Appendix 1: Guideline for interviews with physicians

<table>
<tr><td colspan="2"><h2>Introduction</h2></td></tr>
<tr><td colspan="2">Interviewer presents him/herself; Information on recording and pseudonymization; Information on compensation for expenses

Do you have any further questions?

Then we will now begin the interview. In the letter we wrote to you, we estimated that the interview would take 20-30 minutes. I still consider that estimate to be realistic.

This interview concerns error management [...In the first instance, the focus will be on regular practice work, without the special challenges posed by the current pandemic]. As you may know, error management is, at least theoretically, mandatory for practices, but how it is supposed to be carried out has not been clearly specified. We would therefore like to know how you currently apply error management in your practice. What do you do in your practice if something goes wrong? It is important to realize that there are no right and wrong answers – we would simply like you to report on your everyday work.</td></tr>
<tr><td>Prompt</td><td>Further questions</td></tr>
<tr><td colspan="2"><h2>Critical incidents</h2></td></tr>
<tr><td>When you think about critical incidents that might occur in medical practices, what sort of things do you think about?</td><td></td></tr>
<tr><td colspan="2"><h2>Work processes in the practice and discussion within the team</h2></td></tr>
<tr><td>As we just mentioned, error management has not been clearly defined. What do you do in your practice if something goes wrong? (When something doesn’t work out the way it is intended to?)</td><td>- If answer is too general: Can you think of a specific situation?
- If so: describe the situation?
- I would like to ask you a few questions based on the example you gave
- What do you do after the event?</td></tr>
</table>

	- If something happens, do you have the possibility to immediately **document** it in writing?<br>*If yes:* In what way? (perhaps provide examples: do you enter the information into the computer or write it down on paper? Does a form exist for that express purpose, or is the incident described in free-text?)<br>- **Who**, among practice team members, finds about the event?<br>*In case of psychiatric work:*<br>- **Who** finds out about the incident? Who do you discuss the event with? *(if team supervision or Balint groups are mentioned, then the interview can be conducted along the lines of a team meeting)*<br>- **How** do people find out about the incident?<br>- Do **team meetings** take place in your practice?<br>*If yes:* who takes part in them?<br>- Do additional discussions take place that only involve physicians?<br>- **How** do you go about **discussing** such an incident in a team meeting? Can you describe the procedure to me?<br>- When would you say that you have **finished dealing** with the incident?
Are there certain **cases** that you **deal with differently** to those you have just described (are there differences in the way you deal with specific incidents?)	- **What** do you do that **differs** from the procedure you have just described?<br>- What do you do **after the incident**?<br>- When something has happened, do you have the opportunity to **document** it in writing?<br>*If yes:* In what form? (do you enter the information into the computer or write it down on paper? Does a form exist for that express purpose, or is the incident described in free-text?)<br>- Of practice team members, **who** finds about the event?<br>*In case of psychiatric work:*<br>- **Who** finds out about the incident? Who do you discuss the event with? *(if team supervision or Balint groups are mentioned, then the interview can be conducted along the lines of a team meeting)*

	- **How** do people find out about the incident?<br>- *If team meetings are mentioned:*<br>**Who** actually takes part in team meetings? Do additional meetings take place for doctors only?<br>- **How** do you go about **discussing** such an incident in a team meeting? Can you describe the procedure to us?<br>- When would you say that you have **finished dealing** with the incident?
Have you ever experienced that an error ultimately contributed towards an **improvement** in work processes?	**How** did it come about (that there was an improvement)?
If team meetings are not mentioned: When do you discuss work-related events with other practice team members?	(Handovers, lunch, after work)
Have you ever visited a website such as **jederFehlerzaehlt.de** (every error counts) in order to have a look at anonymous error reports written by other practices?	Have you ever written a **report** yourself?<br><br>*If the webpages have been used for reading rather than for entering a report:* What prevented you from entering a report yourself?
What do you generally use the **internet** for in your practice?	Do all workplaces have **internet access**?
If you could give **young colleagues something to take away on the subject of error management**, what would it be?	
Would you like to comment further on the subject? Is there anything else you would like to add that we haven't spoken about?	
And in conclusion: What influence have developments relating to the Corona virus had on your error management?	How have work processes in your practice changed as a result?
How did you find out about the study?	
